# Challenges in the management of operable triple-negative breast cancer in a survivor of the B-cell acute lymphoblastic leukemia: a case report

**DOI:** 10.3389/fonc.2024.1404706

**Published:** 2024-05-16

**Authors:** Tina Pavlin, Ana Blatnik, Boštjan Šeruga

**Affiliations:** ^1^ Division of Medical Oncology, Institute of Oncology Ljubljana, Ljubljana, Slovenia; ^2^ Faculty of Medicine, University of Ljubljana, Ljubljana, Slovenia; ^3^ Department of Clinical Cancer Genetics, Institute of Oncology Ljubljana, Ljubljana, Slovenia

**Keywords:** triple negative breast cancer, acute lymphoblastic leukaemia, second primary cancer, genetic testing, follow-up, case report

## Abstract

**Background:**

Operable triple-negative breast cancer (TNBC) is an unfavorable subtype of breast cancer, which usually requires an aggressive perioperative systemic treatment. When TNBC presents as a second primary cancer after cured acute leukemia, its management might be challenging.

**Case presentation:**

We present a case report of a young postmenopausal woman with an operable TNBC who had a history of the B-cell acute lymphoblastic leukemia (B-ALL) and graft versus host disease (GVHD) after allogeneic stem cell transplantation (allo-SCT). A history of previous treatment with anthracyclines and radiotherapy and GVHD limited the use of doxorubicin for treatment of her TNBC. Due to the history of GVHD, perioperative treatment with pembrolizumab was omitted. Genetic testing was challenging due to the possible contamination of her tissues with the donor’s cells after allo-SCT. In samples of our patient’s buccal swab, peripheral blood, and tumor tissue, a pathogenic variant in the partner and localizer of *BRCA2* (*PALB2*) gene was found. With neoadjuvant chemotherapy which included carboplatin, a pathologic complete response was achieved. Although our patient has a low risk for recurrence of TNBC, her risk for the development of new primary cancers remains substantial.

**Conclusion:**

This case highlights challenges in the systemic treatment, genetic testing, and follow-up of patients with operable TNBC and other solid cancers who have a history of acute leukemia.

## Background

Triple-negative breast cancer (TNBC) accounts for approximately 15% of all breast cancers and is clinically defined as lacking expression of the estrogen receptor (ER) and progesterone receptor (PR) and overexpression of the human epidermal growth factor receptor (HER) 2. Historically, TNBC has been characterized by an aggressive natural history and worse disease-specific outcomes as compared with other breast cancer subtypes (1). A modern systemic therapy of the operable TNBC includes perioperative chemotherapy (ChT) (i.e., anthracyclines, taxanes with or without carboplatin and capecitabine in patients with residual disease after surgery), an immune checkpoint inhibitor (ICI) pembrolizumab, and an inhibitor of the poly (ADP-ribose) polymerase (PARP) olaparib in patients with breast cancer gene 1 (*BRCA1*) and breast cancer gene 2 (*BRCA2*) pathogenic germline variants (2). When TNBC presents as a second primary cancer after cured acute leukemia, its management might be challenging.

Second primary breast cancers are among the most common second non-skin cancers in survivors of childhood cancers (3). For pediatric patients with an acute lymphoblastic leukemia (ALL), a reported cumulative risk of second primary cancers ranges from 1.2% to 3.3% after 10 to 15 years of follow-up (3–6). More than 80% of ALL results from the clonal proliferation of abnormal B-cell progenitors (B-ALL). ChT for B-ALL consists of induction, consolidation, and long-term maintenance, with central nervous system (CNS) prophylaxis given at intervals throughout therapy. Allogeneic stem cell transplantation (allo-SCT) is a treatment of choice for patients with ALL after first relapse and is also recommended for high-risk patients in the first complete remission (7). Graft versus host disease (GVHD) is a serious and potentially deadly complication of the allo-SCT, which occurs by the donor’s immune effector cells recognizing and destroying the recipient’s tissues and organs, often in the first 3 months after the allo-SCT. After the allo-SCT, 20%–80% of patients develop acute GVHD and 6% to 80% chronic GVHD (8–10). While ALL in children is a highly curable disease, a long-term survival rate in adults with ALL is 30%–45% (7, 11, 12).

Here, we present a case which highlights challenges in the systemic treatment, genetic testing, and follow-up of a patient with an operable TNBC and a history of B-ALL.

## Case report

**Figure 1:**
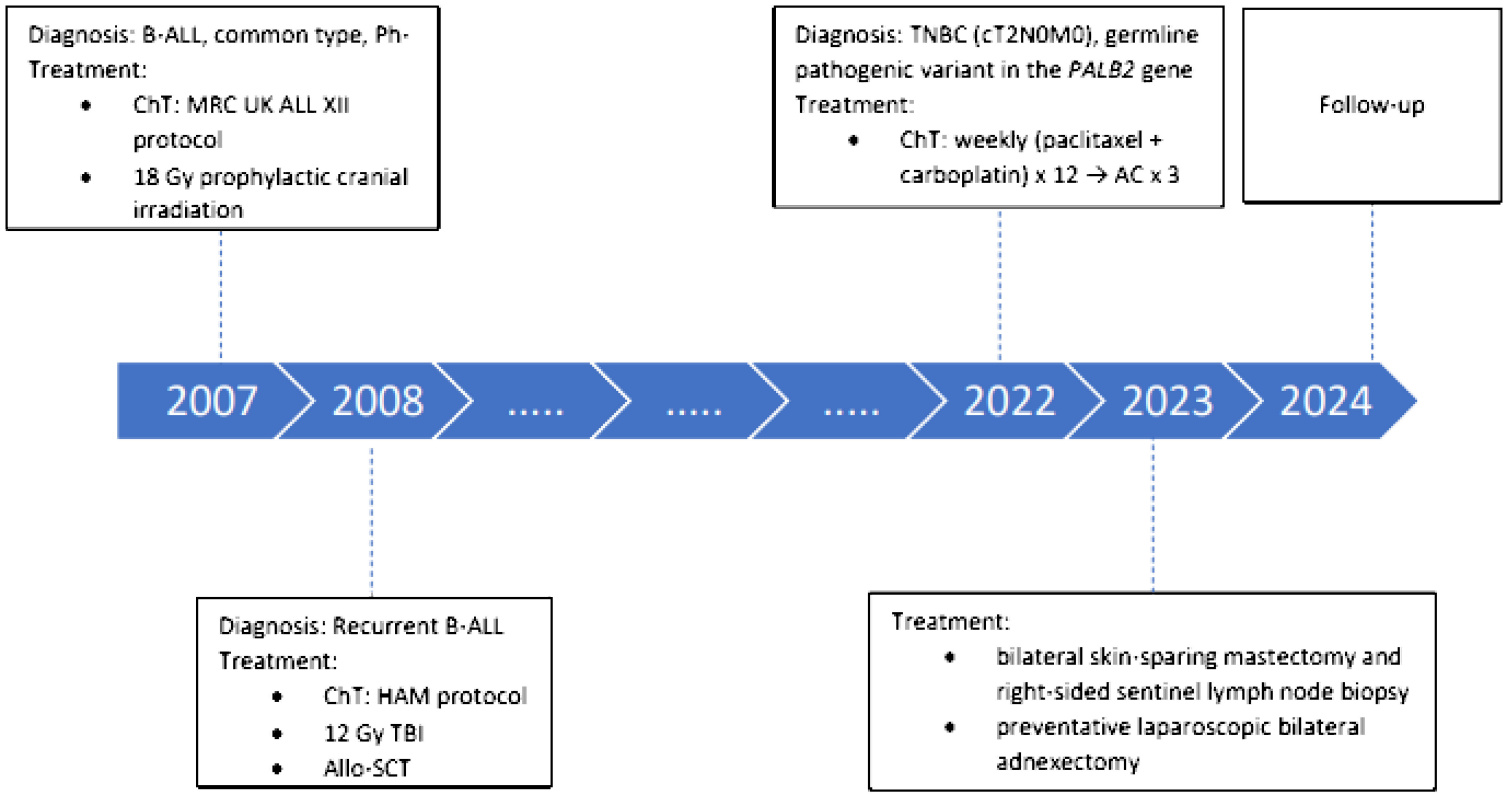
Timeline of the patient’s diagnosis and treatment process. AC, adriamycin, cyclophosphamide; B-ALL, B-cell acute lymphoblastic leukemia; ChT, chemotherapy; HAM, high-dose cytosine arabinoside and mitoxantrone; MRC UK, Medical Research Council United Kingdom; PALB2, partner and localizer of *BRCA2*; Ph-, Philadelphia chromosome-negative; TBI, total body irradiation; TNBC, triple- negative breast cancer.

A timeline of the patient’s diagnosis and treatment process is presented in **Figure 1**. A 40-year-old postmenopausal woman presented with a palpable lump in her right breast in 2022. Mammography showed a suspicious tumor mass of 18 × 20 mm in the upper inner quadrant. She underwent a core needle biopsy, and the breast pathologist reported invasive ductal carcinoma, G3, ER 0%, PR 0%, HER 2 negative, and Ki-67 70%–80%. Computed tomography scan of the thorax and abdomen and bone scan did not show distant metastases. Magnetic resonance imaging (MRI) of the right breast showed a mass sized 21 × 14 mm without involvement of the axillary lymph nodes (cT2 N0 M0, stage IIA). Her family history revealed that her maternal grandmother had an abdominal cancer at the age of 81, one maternal cousin had a tonsil cancer at the age of 57, another maternal cousin had a buccal mucosa cancer at the age of 56, her maternal aunt had a lung cancer at the age of 77, and her paternal uncle had a rectosigmoid cancer at the age of 65. She had a menarche at the age of 14 and went into an iatrogenic menopause after treatment of her B-ALL at the age of 26. She was gravity and parity 0 and never took any hormonal therapy. She had no history of smoking and drinking alcohol but a known allergy to vancomycin. Her history was significant for hyperthyroidism, which was treated with radioiodine and for B-ALL.

In 2007, at the age of 25, our patient was diagnosed with B-ALL, common type, Philadelphia chromosome negative. She was treated with ChT according to the Medical Research Council United Kingdom ALL XII ChT protocol which also included daunorubicin. She also received 18-Gy prophylactic cranial irradiation. At that time, our patient rejected treatment with an allo-SCT after induction treatment. In 2008, her B-ALL relapsed and she received ChT according to the high-dose cytosine arabinoside and mitoxantrone (HAM) protocol and myeloablative 12-Gy total body irradiation (TBI) followed by the allo-SCT. The donor was her mother who was blood type compatible but HLA incompatible. In 2009, she was diagnosed with acute and chronic intestinal, hepatic, and skin GVHD, and for a while she was on and off corticosteroids. At the time of diagnosis of her breast cancer, her B-ALL was in complete remission and she did not have any symptoms of the GVHD. During treatment of the B-ALL, she received a cumulative dose of 120 mg/m^2^ of daunorubicin and 30 mg/m^2^ of mitoxantrone, which is altogether equivalent to 220 mg/m^2^ of doxorubicin.

A multidisciplinary breast cancer tumor board recommended neoadjuvant systemic treatment followed by mastectomy with or without adjuvant systemic treatment. Her baseline echocardiogram was normal. The plan was to treat her with neoadjuvant ChT, consisting of 12 weekly applications of paclitaxel (80 mg/m^2^) and carboplatin (area under the curve [AUC] 1.5), followed by three instead of four cycles of the dose-dense doxorubicin (60 mg/m²) and cyclophosphamide (600 mg/m²) to not to exceed the overall cumulative dose of doxorubicin of 400 mg/m^2^. A consulting hematologist did not advise against the coadministration of the granulocyte-colony stimulating factor (G-CSF) during ChT and treatment with pembrolizumab. After only 4 weeks of neoadjuvant ChT with paclitaxel and carboplatin, primary tumor was not palpable anymore and 7 weeks later, MRI showed a complete radiologic response. Due to the excellent response of primary tumor to ChT and a history of GVHD, treatment with pembrolizumab was omitted. At this point, a multidisciplinary tumor board recommended a continuation of the planned treatment with dose-dense doxorubicin and cyclophosphamide and G-CSF followed by surgery. The last dose of ChT was reduced due to the symptomatic anemia. Otherwise, our patient tolerated treatment with ChT very well. Because of the young age and TNBC pathology, she was referred to a geneticist. Genetic testing for a hereditary breast cancer using a multigene panel was performed using a buccal swab and peripheral blood. Both samples were positive for a heterozygous pathogenic variant in the partner and localizer of the *BRCA2* (*PALB2)* gene [c.1451T>A p. (Leu484*)]. Since she was after allo-SCT and there was a risk for contamination with the donor’s cells, a genetic analysis of the primary breast tumor was performed, which confirmed a pathogenic variant in the *PALB2* gene with a variant allele frequency of 68%. A germline pathogenic variant in the *PALB2* gene was suspected, and therefore she underwent a bilateral skin-sparing mastectomy and right-sided sentinel lymph node biopsy with immediate reconstruction with implants. As our patient was already postmenopausal, she also opted for the immediate preventative laparoscopic bilateral adnexectomy. The postoperative period was uneventful. With neoadjuvant ChT, a pathologic complete response (pCR) was achieved. More than 1 year after surgery, our patient is well and free of cancer.

## Discussion

Patients with operable TNBC usually require multidisciplinary treatment with neo/adjuvant systemic therapy, surgery, and adjuvant radiotherapy to decrease a risk of recurrence and death due to the breast cancer (12, 13). There may be some additional challenges in the management of patients with early TNBC who were previously treated for B-ALL.

Firstly, a risk for the development of cardiac toxicity after treatment of TNBC may be substantially increased in patients who were previously treated for B-ALL. Based on the literature, a recommended maximum lifetime cumulative dose of doxorubicin is 550 mg/m^2^, or, in patients who had received previous mediastinal radiation, 450 mg/m^2^ (14). The probability of developing congestive heart failure (CHF) is estimated to be around 1% to 2% at a cumulative dose of 300 mg/m^2^, and thereafter, a risk for the development of CHF increases steeply (3% to 5% at 400 mg/m^2^; 5% to 8% at 450 mg/m^2^, and 6% to 20% at 500 mg/m^2^) (15, 16). Furthermore, radiotherapy is another known substantial risk factor for the development of cardiovascular (CV) disease (17, 18). Additionally, patients who receive allo-SCT have a 2.3-fold higher risk of the premature CV death (19). As compared with autologous recipients, recipients of the allo-SCT have a higher incidence of long-term CV events (20). Treatment of GVHD includes the use of immunosuppressants, including corticosteroids, leading to a higher prevalence of risk factors for CV disease such as dyslipidemia, hypertension, and insulin resistance (21, 22). In the allo-SCT survivors, chest radiation prior to transplantation is associated with a 9.5-fold increase in the development of coronary artery disease (21, 22). Altogether, our patient received an equivalent of 220 mg/m^2^ of doxorubicin and 12-Gy TBI before the allo-SCT and was later also treated for GVHD. To minimize a risk for the development of heart disease, our plan was not to exceed a cumulative dose of 400 mg/m^2^ of doxorubicin. Instead of the full dose of 240 mg/m^2^ (4 × 60 mg/m^2^), our patient received 162 mg/m^2^ of doxorubicin for her TNBC. However, an anthracycline-free ChT regimen containing carboplatin and paclitaxel/docetaxel might also be a valid treatment option in our patient (23, 24). The G-CSF usage allows administration of higher cumulative doses of ChT and better survival rates, which may both be associated with a higher occurrence rate of second cancers (25). According to the results of systematic review, G-CSF increases a risk for the development of acute myeloid leukemia and myelodysplastic syndrome but not for the ALL (25). Evidence also suggests that G-CSF does not increase a risk for the development of GVHD (26). We conclude that administration of G-CSF is safe in patients with a history of B-ALL.

Secondly, use of ICIs after allo-SCT may increase a risk for the development of GVHD. A contemporary systemic treatment of patients with an operable TNBC now beside ChT also includes an ICI pembrolizumab. In the KEYNOTE 522 phase III study, an addition of pembrolizumab to the neoadjuvant ChT with paclitaxel, carboplatin, doxorubicin, and cyclophosphamide resulted in a higher pCR rate (64.8% vs. 51.2%) and improvement in the event-free but not overall survival (27). However, studies showed that in patients with various relapsed hematologic malignancies who were previously treated with allo-SCT, treatment with ICIs was highly efficacious but also increased a risk for the development of GVHD (14% acute, 9% chronic), including GVHD-related deaths (28). A history of GVHD and a short time interval between the allo-SCT and treatment with an ICI are both associated with a higher risk for the development of GVHD. A risk for the development of GVHD remains increased for several months after treatment with an ICI and is higher when combinations of ICIs are used (29–31). A time interval between the allo-SCT and the diagnosis of TNBC in our patients was long (i.e., 14 years), but data on the safety of ICIs in such cases are still lacking. Due to the history of GVHD and the excellent response to ChT, treatment with pembrolizumab was omitted in our patient. Oncologists should be aware that treatment with an ICI can lead to devastating complications related to GVHD in patients who previously received allo-SCT for hematologic malignancy.

Thirdly, testing for hereditary cancer may be challenging in patients with a history of allo-SCT. When dealing with a young patient with TNBC, genetic testing is of great importance, due to the fact that up to 40% of the early-onset and/or familial TNBC have germline pathogenic variants in *BRCA1*, *BRCA2*, *PALB2*, and some other genes (32). Accordingly, our patient was referred to a geneticist, but since she was an allo-SCT recipient, and her mother was a donor, choosing the most appropriate and reliable biological sample for genetic testing proved difficult (33, 34). After the allo-SCT, not only blood cells but also other cell subtypes may be replaced by cells of donors’ origin during a process called adult stem cell plasticity phenomenon (33). Bone marrow and peripheral blood stem cells have a potential to transdifferentiate or dedifferentiate into neural, bone, muscular, cartilage, liver, gut, alveolar, buccal, epidermal, or endothelial cells, and these exogenous cells can represent between 0.1% and 10% of tissue-specific cells after allo-SCT. However, it has been shown that hair follicle cells lack adult stem cell plasticity and they remain of the recipient’s origin for more than 20 years after the allo-SCT (33). The best biological sample for genetic testing is still being debated, but the National Comprehensive Cancer Network guidelines recommend that DNA of allo-SCT recipients should be extracted from the skin fibroblasts, hair follicles, or other non-hematopoietic origin tissue of the allo-SCT recipients. When this is not possible, buccal swab can be considered as an appropriate alternative source of DNA even though buccal epithelial cells can be replaced by donor-derived cells (33–35). In our case, patient’s buccal swab, peripheral blood and tumor tissue were tested, and all samples were positive for a pathogenic variant in the *PALB2* gene (c.1451T>A p. (Leu484*)). The variant allele frequency in tumor tissue was high, suggesting that our patient’s TNBC developed predominantly due to the *PALB2* variant; however, previous management of her B-ALL could also have contributed to the development of TNBC. Considering that the patient’s blood sample presumably consisted of donor cells, genetic testing might have incidentally identified her mother as a carrier of the *PALB2* pathogenic variant. As her mother has not undergone genetic testing due to her advanced age, her carrier status cannot be verified. As testing for *PALB2* variants can be important for cancer risk assessment and screening as well as pregnancy planning, our patient’s maternal relatives were offered genetic counselling, but they have not responded to the invitation so far. This case highlights a possibility that genetic testing performed after allo-SCT might reveal pathogenic variants of donor’s origin, which might have clinical implications for the donor (36). A *PALB2*-variant breast cancer is usually associated with an aggressive clinicopathological features and is often of triple-negative phenotype (37). PALB2 protein participates in a process of the homologous recombination, and there is evidence that rapid and durable responses could be achieved with a platinum-based chemotherapy in *PALB2-*associated breast cancers (37). In our patient, a combination of carboplatin and paclitaxel resulted in a rapid complete clinical response of the primary tumor, which is in line with previous reports.

Finally, in children and young adults who are treated for various hematologic malignancies, it is important to consider a risk for new primary cancers later in life. Our case also indicates that among patients who develop a second primary solid cancer after hematologic malignancy, there is a subset of patients who have a hereditary cancer. It is well known that biallelic pathogenic variants in the *PALB2* gene result in a subtype of Fanconi anemia, whereas the monoallelic pathogenic variant in *PALB2* predisposes carriers to different cancers such as breast, pancreatic, and ovarian cancers (38). In carriers of the pathogenic variant in the *PALB2* gene, a lifetime risk for the development of breast cancer is 40%–60%, for ovarian cancer 3%–5%, and for pancreatic cancer 2%–3%. Guidelines of the European Society of Medical Oncology recommend that women with pathogenic variants in the *PALB2* should have clinical breast examination every 6–12 months at age 20–25 years, annual MRI at age 20–29 years, and annual breast MRI and/or mammogram at age 30–75 years; they should also consider a risk-reducing mastectomy and adnexectomy. Screening for pancreatic cancer with an annual MRI and/or endoscopic ultrasound from the age of 50 (or 5–10 years younger than the affected relative) can also be considered, when at least one first- or second-degree, presumably *PALB2*-positive relative develops exocrine pancreatic cancer (39). Due to pathogenic variant in *PALB2* our patient underwent bilateral skin-sparing mastectomy and preventative bilateral adnexectomy and is now in the follow-up program in our cancer center. After mastectomy, there was no need for adjuvant irradiation, which could increase a risk for the development of new primary breast cancer in the case of breast-conserving surgery. Fortunately, our patient achieved a pCR and has a very low risk for the recurrence of TNBC. However, after intensive treatment with ChT and radiotherapy, which she received for her B-ALL and TNBC and a known pathogenic variant in the *PALB2* gene, her risk for the development of new primary cancers other than breast and ovarian cancers, particularly pancreatic cancer, may be substantial. Pancreatic cancer surveillance is a contentious subject, with controversies regarding the identification of high-risk individuals, imaging methods, screening intervals, and patient outcomes. In the future, patients such as ours will hopefully benefit from personalized risk assessment and additional blood-based as well as radiomic biomarkers, including the use of artificial intelligence (40). An annual whole-body MRI might also prove useful for this patient, considering her risk of other new primary cancers.

## Conclusions

To our knowledge, this is a first published report which comprehensively highlights the complexity of management of a patient with a second primary TNBC after cured acute leukemia, which was also treated with an allo-SCT. A potential benefit of systemic anticancer therapy should be carefully balanced against its possible harms in patients with a second primary TNBC. Additionally, in patients with a history of allo-SCT, genetic testing for a hereditary cancer may be challenging. Patients with a second primary solid cancer and a history of hematologic malignancy, especially those with a known hereditary cancer, have a substantial risk for new primary cancers. Future research should focus on the development of optimal personalized follow-up programs in this population of patients. Furthermore, development of effective new systemic therapies (e.g., next-generation immunotherapy and targeted agents) which in contrast to ChT do not substantially increase a risk for new primary cancers would be of great importance especially for young patients with TNBC and other solid cancers, which have a complicated treatment history.

## Ethics statement

Written informed consent was obtained from the individual(s) for the publication of any potentially identifiable images or data included in this article.

## Author contributions

TP: Conceptualization, Investigation, Resources, Writing – original draft. AB: Conceptualization, Investigation, Writing – review & editing. BŠ: Conceptualization, Funding acquisition, Investigation, Supervision, Writing – review & editing.
